# A multi-institutional retrospective cohort of adult-onset medulloblastoma in the modern era

**DOI:** 10.1093/noajnl/vdae231

**Published:** 2025-01-22

**Authors:** Alipi V Bonm, Michael S Rutenberg, Kate E Therkelsen, John Herbst, Anurag Sanaf, Marissa A Sherwood, John Y Rhee, Tresa M McGranahan, Patrick J Cimino, L Nicolas Gonzalez Castro, Derek S Tsang, Matthias A Karajannis, Seema Nagpal, Robert J Amdur, Helen Shih, Jason Barber, Lynne P Taylor

**Affiliations:** Ben and Catherine Ivy Center for Advanced Brain Tumor Treatment, Swedish Neuroscience Institute, Seattle, Washington, USA; Department of Radiation Oncology, Mayo Clinic, Jacksonville, Florida, USA; Department of Neurology, Stanford University, Stanford, California, USA; Department of Medical Oncology, Allegheny Health Network Cancer Institute, Pittsburgh, Pennsylvania, USA; Department of Radiation Oncology, Dana Farber Cancer Institute, Boston, Massachusetts, USA; Radiation Medicine Program, Princess Margaret Cancer Centre, University Health Network, Toronto, Canada; Department of Neurology, Brigham and Women’s Hospital, Harvard Medical School, Boston Massachusetts, USA; Division of Adult Palliative Care, Department of Psychosocial Oncology and Palliative Care, Dana Farber Cancer Institute, Boston, Massachusetts, USA; Division of Neuro-Oncology, Department of Medical Oncology, Dana Farber Cancer Institute, Boston, Massachusetts, USA; Department of Neurology, Scripps Medical Center, Encinitas, California, USA; Neuropathology Unit, Division of Intramural Research, National Institutes of Health, Bethesda, Maryland, USA; Department of Neurology, Brigham and Women’s Hospital, Harvard Medical School, Boston Massachusetts, USA; Division of Neuro-Oncology, Department of Medical Oncology, Dana Farber Cancer Institute, Boston, Massachusetts, USA; Radiation Medicine Program, Princess Margaret Cancer Centre, University Health Network, Toronto, Canada; Department of Pediatrics, Memorial Sloan Kettering Cancer Center, New York, New York, USA; Department of Neurology, Stanford University, Stanford, California, USA; Department of Radiation Oncology, University of Florida, Gainesville, Florida, USA; Department of Radiation Oncology, Massachusetts General Hospital, Boston, Massachusetts, USA; Department of Neurological Surgery, University of Washington, Seattle, Washington, USA; Departments of Neurology and Neurosurgery, Alvord Brain Tumor Center, University of Washington, Seattle, Washington, USA

**Keywords:** adjuvant chemotherapy, adult medulloblastoma, concurrent chemotherapy, craniospinal radiation, proton

## Abstract

**Background:**

Adult onset medulloblastoma (aMB) is a rare tumor with limited available evidence. We present a large multi-institutional retrospective cohort of aMB patients treated in the modern era, with an emphasis on understanding the role of chemotherapy at initial diagnosis.

**Methods:**

We included 267 consecutive patients with aMB treated at 7 different institutions from 2000-present, controlling for chemotherapy regimen and cycles received.

**Results:**

Treatment factors were highly intercorrelated with one another and with treating institution. Concurrent chemotherapy was not associated with overall survival (OS). Adjuvant chemotherapy was associated with OS on univariable analyses (HR = 0.55, *P* = .029) and on multivariable analysis when adjusting for risk status (HR 0.55, *P* = .026) but not when also adjusting for treating institution. Proton craniospinal irradiation was associated with improved survival on univariable (HR = 0.50, *P* = .019) and multivariable analysis adjusting for risk status (HR = 0.51, *P* = .024) but not when treating institution was also considered. On subgroup analysis, adjuvant chemotherapy was associated with improved survival in M0 (HR = 0.55, *P* = .043) but not M1 disease, in patients with subtotal resection (HR = 0.43, *P* = .048) but not those with GTR. Similarly, progression-free survival was improved with chemotherapy in patients with M0 (HR = 0.57, *P* = .032) but not M1 disease, and in patients with subtotal (HR = 0.50, *P* = .054) but not gross total resection.

**Conclusions:**

There was no benefit of concurrent chemotherapy. Adjuvant chemotherapy was associated with improved overall survival and this effect was driven by select subgroups, specifically those with M0 disease and those with residual tumor. We could not confirm that these associations are independent of the treating institution.

**Key Points**

1. There was no benefit of concurrent chemotherapy in adult medulloblastoma.
2. Adjuvant chemotherapy improved survival in patients with M0 disease and with residual tumors.
3. All treatments were highly intercorrelated with each other and institution.

**Importance of the Study**

This is the largest and only multicenter retrospective study of adult medulloblastoma not derived from a national database and with full annotation, representing a cohort reflective of modern treatment practice and with 2 decades of follow-up. We confirm no survival benefit with concurrent chemotherapy and a positive association between adjuvant chemotherapy and survival, including when controlling for risk status as previously described. We extend this finding to show that the association between adjuvant chemotherapy and survival is driven by patients with M0 disease and with residual tumors after surgery. We also show high intercorrelation between treatment factors including use of concurrent and/or adjuvant chemotherapy, CSI dose, protons/photons, and treating institution. Concordantly in multivariate models that account for risk status and treating institution, we do not replicate the association between adjuvant chemotherapy and survival.

Adult-onset medulloblastoma (aMB) is a rare CNS tumor with an incidence of 0.5 per million annually, whereas childhood-onset medulloblastoma accounts for a quarter of intracranial tumors in pediatric patients and an annual incidence of 6 per million.^1,2^ Most aMB tumors are found in young adults in the second and third decade of life and differ from pediatric tumors by both overall incidence and frequency of molecular subtypes. For example, group 3 medulloblastomas, which comprise approximately 28% of pediatric medulloblastomas, are rare in adults. The most common molecular subgroups in adults are sonic hedgehog (SHH), followed by non-SHH/non-WNT (group 4), and then wingless-related integration site (WNT) tumors.^3,4^ Due to the scarcity of data, aMB patients have historically been treated according to protocols previously validated in the pediatric population, despite the widespread belief that aMB is a different disease from the pediatric variant,^5^ as also stated in NCCN. Standard of care treatment comprises surgical resection followed by craniospinal irradiation (CSI) with a posterior fossa boost, based on early reports showing significantly improved 5-year survival in aMB patients receiving >54Gy (91%) vs. < 54Gy (33%) to the posterior fossa.^6^ This same study also identified latency to CSI as a prognostic factor with lower 5-year progression-free survival (PFS) for CSI delayed beyond 6 weeks after surgery.^6^ Uncertainty remains regarding the value of concurrent chemotherapy, typically with vincristine or carboplatin, although this is commonly used in clinical practice.^7^ Pre-radiation chemotherapy with cisplatin, etoposide, cyclophosphamide, and vincristine was studied in a prospective cohort of 11 aMB patients over 6 years and showed shorter than expected OS, suggesting this regimen may cause harm.^8^ Adjuvant chemotherapy after CSI was historically reserved for high-risk patients, based on prospective data from Brandes et al.^9^ Three recent meta-analyses reported an association between chemotherapy treatment and increased survival in aMB,^10–12^ though other retrospective studies did not replicate this finding.^5^ Several of these studies are limited in that they pooled all chemotherapy regimens, did not collect data on number of cycles completed, and did not distinguish between adjuvant and concurrent chemotherapy. The most commonly used chemotherapy regimens include cisplatin or carboplatin with lomustine and vincristine, or etoposide and cyclophosphamide.^13^ There remains a need for aMB-specific data, particularly with respect to prognostic markers and treatment protocols. Here, we present a modern retrospective cohort from 7 institutions with full clinical annotation, with an emphasis on attempting to validate prior findings and assess the association between chemotherapy and overall survival (OS).

## Methods

We collected a retrospective cohort of consecutive adult (age ≥ 18 years at the time of diagnosis) patients with medulloblastoma diagnosed from 2000-present at the following institutions: University of Washington, Stanford University, the University of Florida, Memorial Sloan Kettering Cancer Center, Massachusetts General Hospital, Dana-Farber Cancer Center, and the Princess Margaret Cancer Centre. The study was approved by the respective institutional review boards at all institutions and the requirement for informed consent was waived. All data were derived exclusively from the electronic medical record in this no-contact study. There were no exclusion criteria other than missing records. Of note, the data from the Princess Margaret Cancer Centre^14^ and Massachusetts General Hospital^15^ were previously published. For these institutions, individual patient-level data were contributed by the authors, with additional collection of several data points not included in the original studies.

Overall survival and PFS were calculated starting from the date of surgery, through to either last contact or date of death or hospice placement, or in the case of PFS, to the date of progression as determined by the treating team. The maximal tumor dimension was derived from 3-dimensional measurements in the original radiology report. In cases where this was missing, measurements documented by the neuro-oncologist were used. In some cases, only 2 dimensions were known.

Differences in patient and treatment characteristics by site were tested for univariable statistical significance using Fisher’s exact tests for categorical variables and Kruskal–Wallis tests for continuous and ordinal variables. Differences in mortality and progression were tested for univariable significance using log-rank tests, and for site-adjusted significance using mixed-effects Cox proportional-hazards models modeling site with a random intercept. The strength of the correlations among covariates was assessed using Spearman correlation, Kruskal–Wallis, and Fisher’s exact tests as determined by the nature of the variables involved. Overall and progression-free survival were modeled using mixed-effects Cox proportional-hazards regression with the site represented using a random intercept. Estimates generated from multivariable Cox models were considered reliable when there were at least 7 events-per-variable. A two-sided *P*-value threshold of 0.05 was used to define statistical significance, and no adjustments were made for multiple comparisons. All analyses were carried out using SPSS version 26 and SAS version 9.4 statistical software, with the exception of power analyses which were performed using a calculator available at https://sample-size.net/sample-size-survival-analysis/.

## Results

A total of 267 patients were included in the study from 7 large cancer centers. Population characteristics of the total cohort are presented in Table 1 and are separately broken down by individual institution in Supplementary Table S1. The median age at diagnosis was 28.5 years, patients were 60% male, the median initial Karnofsky Performance Status (KPS) was 80, the mean maximal tumor dimension was 4.1 cm, and 19% of patients in our cohort had Chang Stage M1 or greater disease. We found significant differences in the distribution of several patient characteristics between centers, including age, KPS, histology, and hydrocephalus at diagnosis. Similarly, physician-related variables differed broadly between institutions, including the rate of VP shunting, use of photon or proton radiation, craniospinal dose, use of concurrent and adjuvant chemotherapy, and type of chemotherapy used.

**Table 1.** Patient characteristics. Abbreviations used: IQR = interquartile range, VPS = ventriculoperitoneal shunt. ^1^Statistical significance of each variable by logrank test. ^2^Cox regression adjusting for “site” as a random effect.

	Overall (*N* = 267)	Mortality		Progression	
		10-year OS	*P* ^1^ *P* ^2^	10-year PFS	*P* ^1^ *P* ^2^
**Subjects**	**267**	69.6%		61.4%	
**Age at Dx**					
Median (IQR)	28.5 (23–36.5)		.672 .296		.120 .134
≤24	91 (34%)	77.9%		71.5%	
25–33	92 (34%)	64.9%		51.3%	
34+	84 (31%)	63.6%		59.5%	
**Sex**					
Male	160 (60%)	62.0%	.**023** .**023**	55.1%	.065 .092
Female	107 (40%)	80.6%		70.9%	
**KPS at Dx**					
Median (IQR)	80 (70–90)		.188 .179		.578 .147
0–70	76 (31%)	63.0%		61.2%	
71–100	167 (69%)	73.2%		61.8%	
Unknown	24	67.1%		58.1%	
**Max. diameter**					
Mean (SD)	4.1 (1.2)		.701 .624		.515 .478
<3.7	69 (32%)	78.3%		73.1%	
3.7–4.5	75 (35%)	76.8%		68.0%	
4.6+	72 (33%)	70.2%		54.9%	
Unknown	51	57.6%		52.9%	
**Chang M-stage**					
M0	211 (81%)	70.4%	.248 .201	62.4%	.341 .339
M1-3	49 (19%)	62.6%		56.0%	
Unknown	7	31.3%		66.7%	
**Histology**					
Classic	129 (53%)	67.9%	.886 .937	65.9%	.293 .221
Nodular/desmoplastic	81 (33%)	69.3%		60.0%	
Large cell/anaplastic	32 (13%)	76.8%		60.9%	
Unknown	25	70.3%		49.3%	
**Group**					
WNT	9 (11%)	83.3%	.850 .822	83.3%	.276 .303
SHH	60 (71%)	46.8%		36.7%	
Group 4 (non-WNT/Non-SHH)	16 (19%)	79.5%		83.0%	
Unknown	182	16.5%		15.9%	
**Best EOR pre-RT**					
Biopsy or subtotal	84 (31%)	58.0%	.057 .109	52.3%	.108 .157
Gross total	183 (69%)	75.4%		65.6%	
**Risk status**					
Standard	156 (58%)	73.9%	.302 .382	64.8%	.254 .276
High	111 (42%)	63.8%		56.7%	
**Hydrocephalus**					
No	114 (46%)	66.0%	.254 .277	61.6%	.837 .879
Yes	136 (54%)	77.2%		64.0%	
Unknown	17	47.3%		39.2%	
**VPS**					
No	191 (78%)	67.1%	.256 .120	59.7%	.364 .298
Yes	55 (22%)	81.9%		68.5%	
Unknown	21	59.1%		53.5%	
**Latency to RT (days)**					
Mean (SD)	44.3 (33.1)		.555 .607		.970 .908
<42	160 (63%)	71.5%		62.5%	
≥42	92 (37%)	69.5%		62.2%	
Unknown	15	46.8%		45.5%	
**Radiation**					
Photon	139 (53%)	62.1%	**.017** .160	57.0%	.172 .712
Proton	121 (47%)	83.9%		70.5%	
Unknown	7	53.3%		40.0%	
**CSI dose**					
≥30Gy	182 (78%)	67.3%	.164 .613	58.6%	.005 .012
<30Gy	52 (22%)	89.3%		89.2%	
Unknown	33	49.6%		22.7%	
**Concurrent chemo**					
No	171 (64%)	63.0%	.157 .604	55.8%	.148 .399
Yes	96 (36%)	82.8%		70.2%	
**Concurrent type**					
None	171 (64%)	63.0%	**.041** .211	55.8%	.156 .348
Vincristine	78 (29%)	84.8%		71.1%	
Etoposide	5 (2%)	40.0%		40.0%	
Carboplatin and vincristine	13 (5%)	88.9%		75.5%	
**Adjuvant (any chemo regimens)**					
0	98 (39%)	59.1%	.222 .650	55.6%	.359 .545
1–2	37 (15%)	72.9%		65.7%	
≥3	118 (47%)	77.5%		63.6%	
Unknown	14	88.9%		70.0%	
**Adjuvant (standard chemo regimens)**					
0	97 (42%)	59.1%	.113 .432	55.6%	.269 .454
1–2	28 (12%)	63.6%		63.3%	
≥3	107 (46%)	81.7%		64.9%	
Unknown	35	70.4%		64.0%	

**Figure 1.**
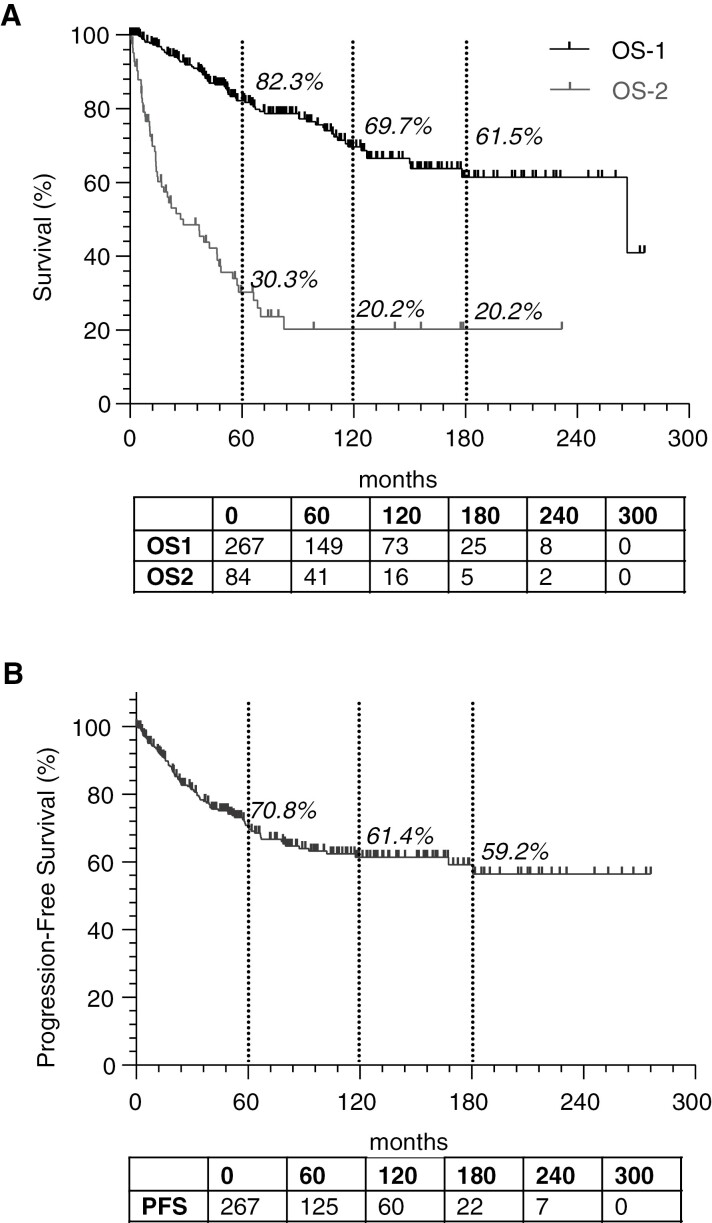
Population statistics. (A) Overall survival is depicted from diagnosis (OS-1) or from time of first progression (OS-2). (B) Progression-free survival from time of diagnosis.

Overall outcomes from the cohort are presented in Figure 1. From initial diagnosis, the 5-year, 10-year, and 15-year overall survival was 82.3%, 69.7%, and 61.5% respectively. Progression-free survival was 70.8% at 5 years (*N* = 149), 61.4% at 10 years (*N* = 73), and 59.2% at 15 years (*N* = 25). In recurrent medulloblastoma, overall survival from the time of first recurrence was 30.3% at 5 years (*N* = 16), and not evaluable at 10 years (*N* = 5) or 15 years (*N* = 2) due to low numbers.

We subsequently performed an analysis of covariate correlations, presented in Table 2. A striking finding was that all factors related to clinical decision-making and treatment selection were highly correlated with one another (see lightly-shaded quadrant in Table 2) and also with treating institutions, reflecting varying institutional practices. Additionally, age at diagnosis and Chang M-stage were highly correlated with all treatment factors, likely reflecting clinical risk stratification. Patients selected for adjuvant chemotherapy were younger, more likely to have metastatic disease, 5-fold more likely to receive concurrent chemotherapy, and started radiation a week earlier than patients who did not receive adjuvant chemotherapy (Supplementary Table S2).

**Table 2.** Covariate correlations. The strength of the correlations among covariates was assessed using Spearman correlation, Kruskal–Wallis, or Fisher’s exact tests depending on the nature of the variables involved. * Indicates categorical variables.

	Inst*	Age	Sex	KPS	MaxDim	MStage	Histo*	Group*	EOR	Risk	Hydrocep	VPS	Latency	Proton	CSI dose	Concurrent	AdjChemo	Std Adj
Inst*	–	**.011**	0.931	^<^ **0.001**	0.228	0.194	**0.032**	0.449	0.305	0.229	**0.010**	**0.046**	0.403	^<^ **0.001**	^<^ **0.001**	^<^ **00.001**	^<^ **00.001**	^<^ **00.001**
Age	**0.011**	–	0.447	**0.003**	0.262	0.909	0.793	0.510	0.026	0.039	**0.003**	0.108	0.067	0.104	^<^ **0.001**	^<^ **00.001**	**0.001**	^<^ **00.001**
Sex	0.931	0.447	–	0.671	0.260	0.246	0.277	0.630	0.152	0.903	0.076	0.788	0.335	0.429	**0.014**	0.692	0.811	0.414
KPS	^<^ **00.001**	**0.003**	0.671	–	0.693	0.599	**0.017**	0.213	0.100	0.407	0.301	**0.041**	0.786	**0.004**	0.090	0.322	0.084	0.063
Max Dim	0.228	0.262	0.260	0.693	–	0.805	0.163	0.240	0.074	0.178	0.106	0.525	0.962	0.680	0.739	0.637	0.600	0.678
M Stage	0.194	0.909	0.246	0.599	0.805	–	0.113	**0.001**	0.025	^<^ **0.001**	0.864	0.538	**0.017**	0.536	**0.001**	0.492	**0.012**	**0.031**
Histo*	**0.032**	0.793	0.277	**0.017**	0.163	0.113	–	**0.001**	0.065	**0.026**	0.299	0.697	0.132	0.108	0.139	0.441	0.936	0.999
Group*	0.449	0.510	0.630	0.213	0.240	**0.001**	**0.001**	–	**0.011**	**0.003**	0.613	0.652	0.560	0.118	0.915	0.912	0.070	0.170
EOR	0.305	**0.026**	0.152	0.100	0.074	**0.025**	0.065	**0.011**	–	^<^ **0.001**	0.078	0.558	0.055	0.267	**0.001**	0.250	0.610	0.689
Risk	0.229	**0.039**	0.903	0.407	0.178	^<^ **0.001**	**0.026**	**0.003**	^<^ **0.001**	–	0.424	0.323	^<^ **0.001**	0.284	^<^ **0.001**	0.623	0.115	0.207
Hydroceph	**0.010**	**0.003**	0.076	0.301	0.106	0.864	0.299	0.613	0.078	0.424	–	^<^ **0.001**	0.551	0.684	0.230	0.140	**0.024**	**0.016**
VPS	**0.046**	0.108	0.788	**0.041**	0.525	0.538	0.697	0.652	0.558	0.323	^<^ **0.001**	–	0.139	**0.013**	0.141	0.830	0.892	0.729
Latency	0.403	0.067	0.335	0.786	0.962	**0.017**	0.132	0.560	0.055	^<^ **0.001**	0.551	0.139	–	0.097	0.953	0.159	**0.006**	**0.005**
Proton	^<^ **00.001**	0.104	0.429	**0.004**	0.680	0.536	0.108	0.118	0.267	0.284	0.684	**0.013**	0.097	–	^<^ **0.001**	^<^ **00.001**	^<^ **00.001**	^<^ **00.001**
CSI dose	^<^ **00.001**	^<^ **00.001**	**0.014**	0.090	0.739	**0.001**	0.139	0.915	0.001	^<^ **0.001**	0.230	0.141	0.953	^<^ **0.001**	–	^<^ **00.001**	^<^ **00.001**	^<^ **00.001**
Concurrent	^<^ **00.001**	^<^ **00.001**	0.692	0.322	0.637	0.492	0.441	0.912	0.250	0.623	0.140	0.830	0.159	^<^ **0.001**	^<^ **0.001**	---	^<^ **00.001**	^<^ **00.001**
Adj Chemo	^<^ **00.001**	**0.001**	0.811	0.084	0.600	**0.012**	0.936	0.070	0.610	0.115	**0.024**	0.892	**0.006**	^<^ **0.001**	^<^ **0.001**	^<^ **00.001**	---	---
Std Adj	^<^ **00.001**	^<^ **00.001**	0.414	0.063	0.678	**0.031**	0.999	0.170	0.689	0.207	**0.016**	0.729	**0.005**	^<^ **0.001**	^<^ **0.001**	^<^ **0.001**	---	---

Abbreviations used: Inst = institution; KPS = Karnofsky performance status, MaxDim = maximal dimension, ChangM = Chang M-stage, Histo = histology; Mgroup = molecular group, EOR = extent of resection, Hydro = hydrocephalus; VPS = ventriculoperitoneal shunt; CSI = craniospinal irradiation; Conc = concurrent chemotherapy; Adj Chemo = any adjuvant chemotherapy; Std Adj = standard adjuvant chemotherapy regimens.

We performed univariable (Table 3) analyses for both OS and PFS looking at both patient and treatment variables. We found a significant effect of treating institutions on both OS (*P* = .022) and PFS (*P* = .047). There was a nonsignificant trend towards improved survival for patients with GTR (HR = 0.62, *P* = .059) and significantly improved survival for patients receiving proton radiation (HR = 0.50, *P* = .019), but neither of these factors was associated with improved PFS. Low-dose CSI was associated with improved PFS, reflecting accurate clinical risk stratification. There was no effect of concurrent chemotherapy on either OS or PFS. To understand the role of adjuvant chemotherapy, we removed all non-standard chemotherapy regimens, standard regimens were defined as a platinum agent (carboplatin or cisplatin) with lomustine and with or without vincristine, or cyclophosphamide and etoposide. Examples of non-standard adjuvant regimens that were removed for this analysis include temozolomide monotherapy, methotrexate-based therapy (either monotherapy or with procarbazine and vincristine), vincristine/etoposide/procarbazine, and etoposide monotherapy. Patients receiving adjuvant chemotherapy had improved OS (HR = 0.55, *P* = .029). Similarly, a trend was observed towards improved PFS (HR = 0.67, *P* = .089). When considering only patients receiving 3 or more cycles of a standard chemotherapy regimen were included, we again observed a significant association with OS (HR = 0.57, *P* = .048) and a trend towards improved PFS (HR = 0.69, *P* = .119).

**Table 3.** Univariable analyses. Statistical significance by log-rank test.

Variable	Overall survival		Progression-free survival	
	HR	*P*	HR	*P*
**Site** (Random effect)	–	.**022**	–	.**047**
**Age** (per + 10 y)	1.19 (0.94, 1.50)	.140	1.20 (0.98, 1.47)	.*080*
**KPS** (per + 10)	0.88 (0.73, 1.07)	.208	0.93 (0.78, 1.10)	.399
**M-Stage** (≥ 1 vs 0)	1.40 (0.79, 2.48)	.250	1.29 (0.76, 2.18)	.343
**EOR** (GTR vs STR/Bx)	0.62 (0.38, 1.02)	.*059*	0.70 (0.45, 1.08)	.110
**Risk status** (High vs standard)	1.30 (0.79, 2.13)	.303	1.28 (0.84, 1.97)	.255
**Hydrocephalus** (Yes vs no)	0.74 (0.43, 1.25)	.256	0.95 (0.60, 1.50)	.836
**VP shunt** (Yes vs no)	0.67 (0.34, 1.34)	.260	0.77 (0.44, 1.36)	.365
**Latency to RT** (≥ 42 vs < 42)	0.85 (0.49, 1.47)	.556	1.01 (0.64, 1.60)	.970
**Radiation** (Proton vs photon)	0.50 (0.28, 0.89)	.**019**	0.73 (0.46, 1.15)	.174
**CSI dose** (< 30Gy vs ≥ 30Gy)	0.57 (0.26, 1.27)	.170	0.32 (0.14, 0.74)	.**007**
**Concurrent chemo** (Yes vs no)	0.68 (0.39, 1.17)	.160	0.71 (0.45, 1.13)	.150
**Adjuvant chemo** (Any vs none)	0.55 (0.33, 0.94)	.**029**	0.67 (0.42, 1.06)	.*089*
**Adjuvant chemo** (3 + cycles vs none)	0.57 (0.33, 1.00)	.**048**	0.69 (0.43, 1.10)	.119

Multivariable analysis was performed (Table 4) to control for risk status, according to the same method reported by Majd et al.^12^ Given the strong intercorrelation between treatment decisions and treating institution (Table 2), we also added a second analysis to control for both risk status and treating institution. We reproduced a significant association between proton CSI and OS (HR = 0.51, *P* = .024) when adjusting for risk status, but this effect was not maintained when adjusting for both risk status and treating institution. Similarly, we confirmed the association between standard adjuvant chemotherapy (HR = 0.57, *P* = .047) and ≥ 3 cycles of standard chemotherapy (HR = 0.57, *P* = .047), although neither of these effects were maintained when adding treating institution as a cofactor. There was a trend towards longer PFS in both the group receiving any and ≥ 1 (HR = 0.66, *P* = .072) and ≥ 3 cycles (HR = 0.68, *P* = .107) of standard chemotherapy.

**Table 4.** Multivariable analysis. Statistical significance by Cox regression; with adjustment for risk status and institution as random effects as noted; no adjustment for multiple comparisons. Four mulitvariable models were run for each variable in the first column, adjusting either for risk status or both risk status and treating institution, and looking at either OS or PFS.

Variable	Overall survival				Progression-free survival			
	Adjusting for risk status		Adjusting for institution and risk		Adjusting for risk status		Adjusting for institution and risk	
	HR	*P*	HR	*P*	HR	*P*	HR	*P*
**Radiation** (proton vs photon)	0.51 (0.29, 0.92)	.**024**	0.66 (0.35, 1.26)	.212	0.75 (0.47, 1.18)	.207	0.95 (0.57, 1.59)	.842
**CSI dose** (< 30Gy vs > 30Gy)	0.58 (0.26, 1.33)	.199	0.84 (0.35, 2.01)	.692	0.31 (0.13, 0.72)	.**007**	0.32 (0.14, 0.77)	.**010**
**Concurrent chemo** (Yes vs no)	0.69 (0.40, 1.19)	.179	0.87 (0.49, 1.54)	.622	0.72 (0.45, 1.15)	.166	0.82 (0.51, 1.33)	.430
**Adjuvant Chemo** (Any vs not)	0.55 (0.32, 0.93)	.**026**	0.65 (0.35, 1.18)	.154	0.66 (0.41, 1.04)	.072	0.67 (0.39, 1.15)	.144
**Adjuvant chemo** (3 + vs Not)	0.57 (0.33, 0.99)	.**045**	0.67 (0.37, 1.24)	.206	0.68 (0.43, 1.09)	.107	0.72 (0.43, 1.23)	.235

**Figure 2.**
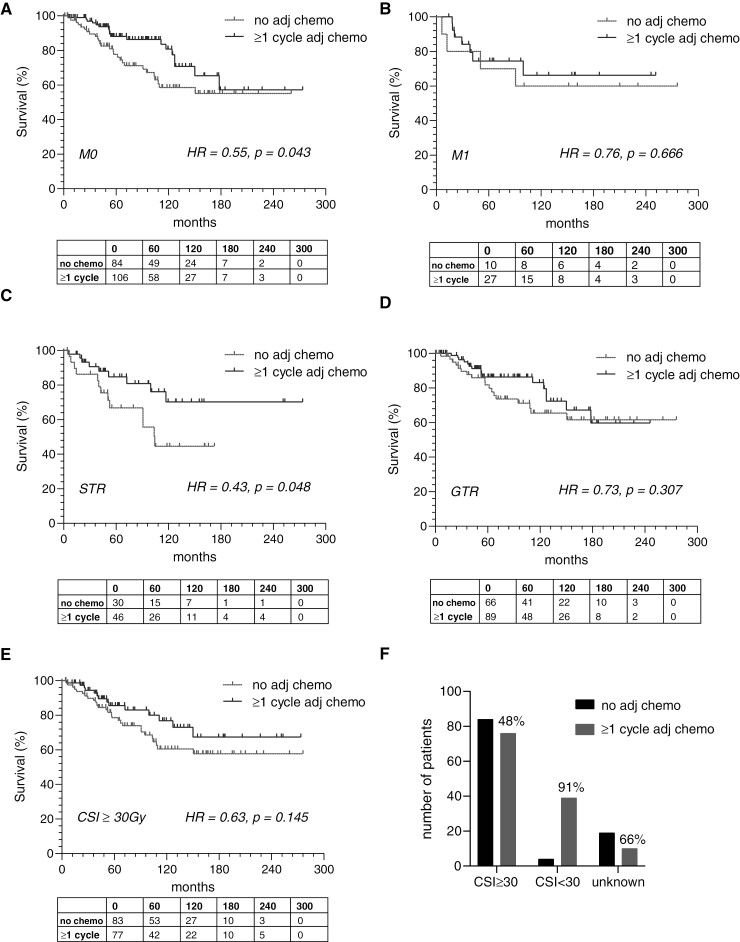
Subgroup analyses. Overall survival was improved with adjuvant chemotherapy in patients with M0 (A) but not M1-3 (B) disease. OS was improved in patients with residual tumor who received adjuvant chemotherapy (C) but unchanged in patients who underwent gross total resection (D). There was a trend towards improved survival in patients treated with standard dose craniospinal radiation (E), but the same analysis could not be performed for patients receiving low dose craniospinal radiation because almost all (91%) of these patients also got adjuvant chemotherapy (F). *P* values are calculated by log-rank test.

We subsequently performed subset analyses (survival is presented in Figure 2, and PFS is presented in Supplementary Figure S1) to better understand treatment in specific subpopulations. Patients with M0 disease had improved survival (HR = 0.55, *P* = .043) and PFS (HR = 0.57, *P* = .032) with adjuvant (standard) chemotherapy, but the same effect was not observed in patients with M1-3 disease (OS: HR = 0.76, *P* = .666; PFS: HR = 1.95, *P* = .358). We found improved survival with adjuvant chemotherapy in patients with residual disease (HR = 0.43, *P* = .048), but the trend in patients with GTR was not significant (HR = 0.65, *P* = .205). Again, the same pattern was observed when looking at PFS in response to chemotherapy in patients with STR (HR = 0.50, *P* = .054) and GTR (HR = 0.85, *P* = .597). Lastly, there were no changes in survival or PFS with adjuvant chemotherapy in patients receiving standard dose (≥ 30Gy) CSI. We were not able to assess the effect of chemotherapy in patients receiving low-dose CSI because a low number (only 9%) of these patients did not receive adjuvant chemotherapy. We also found that institutions where proton therapy was available offered adjuvant chemotherapy in 69.3% of cases whereas institutions without proton therapy offered adjuvant chemotherapy in 23.9% of cases (*P* < .001), and this finding was driven predominantly by a single institution. Subgroup analyses by molecular groups are presented in Supplementary Figure S2 but were limited by missing molecular grouping for many patients resulting in low power.

## Discussion

Adult-onset MB is a rare and challenging disease with limited clinical evidence available to guide management. Here, we analyze a large retrospective multi-institutional series with full clinical annotation from a modern cohort reflective of current treatment practices. Demographics and outcomes in our cohort are comparable with those of smaller available cohorts. Our primary aim for this study was to provide insights regarding the use of chemotherapy in newly diagnosed adult-onset medulloblastoma. We were able to replicate previous findings showing an association between adjuvant chemotherapy use and survival. Additionally, we find that adjuvant chemotherapy is associated with improved survival and delayed progression in patients with M0 but not M1-3 disease, and in patients with residual tumor. On multivariable analysis, we replicated the findings of Majd et Al.,^12^ specifically shows an association between adjuvant chemotherapy and survival when controlling for risk status. We report that treatment teams have a bias towards more aggressive treatment in young and high-risk patients. Additionally, seemingly separate treatment decisions and outcomes including latency to CSI, CSI dose, choice of radiation modality, use of concurrent and of adjuvant chemotherapy—were all highly intercorrelated and correlated with the treating institution, indicating that local practice patterns are a major determinant of treatment selection. When we attempted to control for differing practices across institutions, the association between chemotherapy and survival was not maintained. In contrast, Kann et al. performed a large study with 751 patients from the National Cancer Database and did find an association between adjuvant chemotherapy and survival even when controlling for treating institution. One possible explanation for this is underpowering, although we note that the number of events (deaths) observed in our study was 64 (23.9%), whereas in Kann et al. there were 86 events (18.4%), indicating that the actual difference in power was fairly small between the 2 studies despite their much larger overall cohort size. Univariable power analysis (for the primary analysis) indicated that our dataset was sufficiently powered to detect a hazard ratio of 0.49 with 80% power. The magnitude of the change in HR on multivariable modeling suggested that confounding may also be compromising power. Of the other published retrospective series addressing adjuvant chemotherapy, A meta-analysis of 907 patients by Kocakaya et al. reported an association between adjuvant chemotherapy and OS. However, this study included patients from 1969 onward which implies wide variation in radiation protocols and suggested a benefit to neoadjuvant therapy, which has generally fallen out of favor and other studies^12^ have shown may be harmful, possibly due to delay of radiation. Kocakaya et al. also found a benefit of chemotherapy at recurrence, though a recent study by Gregory et al.^16^ with a larger cohort of recurrent medulloblastoma reported no benefit to chemotherapy. Franceschi et al.^17^ reported a single center retrospective cohort of 48 average-risk aMB patients treated from 1988 to 2016 and observed improved survival for patients treated with any chemotherapy, pooling neoadjuvant, adjuvant, and concurrent regimens. A multivariable analysis was not performed. In contrast, a consortium study of 191 aMB patients treated in the modern era found no association between any chemotherapy (adjuvant or concurrent) and PFS.^5^

We also found an association between proton CSI and OS, though no corresponding association with PFS was observed. This finding was unexpected since proton and photon effects are biologically equivalent. A plausible interpretation of these data would be that both proton and photon CSI offer comparable disease control, but long-term mortality is higher with photon CSI due to higher toxicity compared to protons. Another possibility is that patients with access to protons were selected for higher socioeconomic status which could act as a confounding covariable, as this is a known factor that influences access to proton therapy.^18,19^ We also observed that patients treated with low-dose CSI had improved progression-free survival, likely reflecting accurate risk stratification by the treatment teams, with lower-risk patients chosen to receive low-dose CSI.

An additional strength of our study was the extended follow-up, which allowed us to assess outcomes at 5, 10, and 15 years. After initial diagnosis, 68% of recurrences occur in the first 5 years, 88.8% occur in the first 10 years and none occurred after 15 years, though this was limited by the inclusion of only 25 patients with follow-up beyond 15 years. These data suggest that it may be possible to decrease surveillance frequency after 10 years. After the first recurrence, we found a 30.3% 5-year survival, comparable to Gregory et al.,^16^ but we were underpowered to look at 10-year and 15-year survival.

Importantly, for some analyses included in this study, the results for OS were stronger than those for PFS, which frequently showed the same trends but did not reach significance. An exception was the subgroup analyses, which corresponded very well between OS and PFS. We hypothesize that a potential driver of differences in OS and PFS may be that deaths are specifically captured, encoded, and synchronized across electronic medical records, whereas this is not the case for progressions. Therefore, progression events occurring at an outside institution would have been missed in our data set whereas deaths may have been captured, making OS the more reliable measure in this study. We note that the FDA has specifically called for increased use of OS as a preferred outcome measure, and that prior trials in neuro-oncology and in medical oncology have found discrepancies between PFS and OS, with OS being generally favored as the most reliable and informative outcome measure.^20^

Our study is retrospective in nature and therefore prone to bias. The most important bias to consider is selection bias—specifically that treatment approach was based on perceived risk, in which case a lack of an independent effect of chemotherapy may reflect a positive therapeutic outcome in a group that would have been expected to do more poorly without additional treatment. Another important limitation is that although we included 7 clinical centers, these were all large academic centers, and therefore our cohort was selected against community-based settings and likely has a referral bias. Compared to national database studies, our study is underpowered to detect small effects, though we have shown evidence that it was appropriately powered for the questions of interest. Additionally, we are limited by the lack of central review, either for pathology or for radiographic studies, and by incomplete data on the molecular classification of aMB used in modern practice. As a function of a retrospective cohort, there was extensive missing data, especially with regard to molecular subtypes, as has been observed in similar studies and likely due to the fact that molecular was not routinely performed during the majority of the time window included in the study. Finally, we are limited by considering all-cause mortality, which means that some deaths could have been attributed to causes other than medulloblastoma. These limitations can only be overcome with prospective randomized studies. However, current attempts at prospective trials for adult medulloblastoma are facing major challenges in recruitment and in some cases are facing significant delays, though we also note that at least 1 prospective trial in recurrent aMB enrolled 38 patients before being discontinued for lack of effect.^21^

In summary, we present a large, modern, multicenter retrospective cohort of patients with adult-onset medulloblastoma. We find major differences in practice between institutions and high intercorrelation between treatment factors. We report no effect of concurrent chemotherapy, and an association between adjuvant chemotherapy and survival that is driven specifically by patients with M0 disease and with residual tumor after initial surgery. These effects were not independent of the treating institution.

## Supplementary Material

vdae231_suppl_Supplementary_Tables_S1-S2_Figures_S1-S2

## Data Availability

Deidentified data is available upon request to the corresponding author.
