# Supplementary material for: A multi-institutional retrospective cohort of adult-onset medulloblastoma in the modern era
Source: Neurooncol Adv. 2025 Jan 22;7(1):vdae231. doi: 10.1093/noajnl/vdae231 (PMC11842969; doi:10.1093/noajnl/vdae231)
Supplement: vdae231_suppl_Supplementary_Tables_S1-S2_Figures_S1-S2 [file vdae231_suppl_supplementary_tables_s1-s2_figures_s1-s2.zip › vdae231_suppl_Supplementary_Tables_1-2_Figures_1-2/vdae231_suppl_Supplementary_Tables_1-2_Figures_1-2_legends.docx]

**Table S1: Patient Characteristics by institution.** Statistical significance by Spearman correlation, Kruskal-Wallis, and Fisher's exact tests.

* = categorical variable

|  | **Overall** (N=267) | **Institution** | | | | | | | |
| --- | --- | --- | --- | --- | --- | --- | --- | --- | --- |
|  |  | **1 - UW** (N=57) | **2 - Sta** (N=19) | **3 - UF** (N=50) | **4 - MSK** (N=32) | **5 - Tor** (N=48) | **6 - MGH** (N=58) | **7 - DFCI** (N=8) | **p** |
|  |  |  |  |  |  |  |  |  |  |
| **Subjects** | **267** | **56** | **19** | **47** | **32** | **48** | **57** | **8** |  |
| 10yr Cum.Survival | 69.6% | 70.0% | 69.8% | 62.7% | 70.8% | 48.6% | 91.2% | 85.7% |  |
| 10yr Cum.Progression-Free | 61.4% | 49.4% | 57.1% | 65.2% | 61.1% | 46.5% | 82.2% | 43.8% |  |
|  |  |  |  |  |  |  |  |  |  |
| **Age at Dx** |  |  |  |  |  |  |  |  |  |
| Median (IQR) | 28.5  (23 - 36.5) | 29  (23.5 - 35.75) | 34.5  (29 - 41) | 28.5  (23 - 35) | 33.5  (23.25 - 40.75) | 30.5  (23.25 - 38.75) | 25.5  (21.5 - 31.5) | 25  (21.25 - 26.75) | .**011** |
| ≤24 | 91 (34%) | 19 (34%) | 2 (11%) | 15 (32%) | 10 (31%) | 13 (27%) | 28 (49%) | 4 (50%) |  |
| 25-33 | 92 (34%) | 20 (36%) | 7 (37%) | 19 (40%) | 8 (25%) | 16 (33%) | 18 (32%) | 4 (50%) |  |
| 34+ | 84 (31%) | 17 (30%) | 10 (53%) | 13 (28%) | 14 (44%) | 19 (40%) | 11 (19%) | 0 (0%) |  |
|  |  |  |  |  |  |  |  |  |  |
| **Sex** |  |  |  |  |  |  |  |  |  |
| Male | 160 (60%) | 35 (63%) | 11 (58%) | 26 (55%) | 19 (59%) | 32 (67%) | 32 (56%) | 5 (63%) | .931 |
| Female | 107 (40%) | 21 (38%) | 8 (42%) | 21 (45%) | 13 (41%) | 16 (33%) | 25 (44%) | 3 (38%) |  |
|  |  |  |  |  |  |  |  |  |  |
| **KPS at Dx** |  |  |  |  |  |  |  |  |  |
| Median (IQR) | 80  (70 - 90) | 90  (80 - 90) | 80  (70 - 90) | 80  (70 - 90) | 90  (80 - 90) | 80  (70 - 80) | 80  (70 - 80) | 90  (80 - 90) | ^<^.**001** |
| 0-70 | 76 (31%) | 8 (18%) | 5 (38%) | 23 (49%) | 3 (9%) | 14 (30%) | 22 (41%) | 1 (17%) |  |
| 71-100 | 167 (69%) | 36 (82%) | 8 (62%) | 24 (51%) | 29 (91%) | 33 (70%) | 32 (59%) | 5 (83%) |  |
| Unknown | 24 | 12 | 6 | 0 | 0 | 1 | 3 | 2 |  |
|  |  |  |  |  |  |  |  |  |  |
| **Max. Diameter** |  |  |  |  |  |  |  |  |  |
| Mean (SD) | 4.1 (1.2) | 4.1 (1.2) | 3.9 (1.0) | 3.8 (1.0) | 3.8 (0.8) | 4.4 (1.1) | 4.3 (1.3) | 3.7 (0.8) | .228 |
| <3.7 | 69 (32%) | 14 (26%) | 6 (38%) | 18 (47%) | 7 (33%) | 9 (24%) | 14 (30%) | 1 (25%) |  |
| 3.7-4.5 | 75 (35%) | 21 (40%) | 6 (38%) | 10 (26%) | 10 (48%) | 12 (32%) | 14 (30%) | 2 (50%) |  |
| 4.6+ | 72 (33%) | 18 (34%) | 4 (25%) | 10 (26%) | 4 (19%) | 17 (45%) | 18 (39%) | 1 (25%) |  |
| Unknown | 51 | 3 | 3 | 9 | 11 | 10 | 11 | 4 |  |
|  |  |  |  |  |  |  |  |  |  |
| **Chang M-Stage** |  |  |  |  |  |  |  |  |  |
| Stage 0 | 211 (81%) | 38 (69%) | 14 (88%) | 37 (79%) | 25 (86%) | 41 (85%) | 50 (88%) | 6 (75%) | .211 |
| Stage 1+ | 49 (19%) | 17 (31%) | 2 (13%) | 10 (21%) | 4 (14%) | 7 (15%) | 7 (12%) | 2 (25%) |  |
| Unknown | 7 | 1 | 3 | 0 | 3 | 0 | 0 | 0 |  |
|  |  |  |  |  |  |  |  |  |  |
| **Histology** |  |  |  |  |  |  |  |  |  |
| Classic | 129 (53%) | 24 (52%) | 13 (76%) | 31 (69%) | 17 (55%) | 18 (43%) | 25 (44%) | 1 (25%) | .**033** |
| Nodular/desmoplastic | 81 (33%) | 19 (41%) | 3 (18%) | 12 (27%) | 10 (32%) | 17 (40%) | 19 (33%) | 1 (25%) |  |
| Large cell/anaplastic | 32 (13%) | 3 (7%) | 1 (6%) | 2 (4%) | 4 (13%) | 7 (17%) | 13 (23%) | 2 (50%) |  |
| Unknown | 25 | 10 | 2 | 2 | 1 | 6 | 0 | 4 |  |
|  |  |  |  |  |  |  |  |  |  |
| **Group** |  |  |  |  |  |  |  |  |  |
| WNT | 9 (11%) | 3 (14%) | 1 (13%) | 1 (9%) | 1 (7%) | 0 (0%) | 2 (11%) | 1 (20%) | .681 |
| SHH | 60 (71%) | 16 (73%) | 3 (38%) | 9 (82%) | 12 (80%) | 4 (67%) | 13 (72%) | 3 (60%) |  |
| Group 4 (Non-WNT/ Non-SHH) | 16 (19%) | 3 (14%) | 4 (50%) | 1 (9%) | 2 (13%) | 2 (33%) | 3 (17%) | 1 (20%) |  |
| Unknown | 182 | 34 | 11 | 36 | 17 | 42 | 39 | 3 |  |
|  |  |  |  |  |  |  |  |  |  |
| **Best EOR Pre-RT** |  |  |  |  |  |  |  |  |  |
| Biopsy or subtotal | 84 (31%) | 18 (32%) | 4 (21%) | 19 (40%) | 10 (31%) | 19 (40%) | 12 (21%) | 2 (25%) | .307 |
| Gross total | 183 (69%) | 38 (68%) | 15 (79%) | 28 (60%) | 22 (69%) | 29 (60%) | 45 (79%) | 6 (75%) |  |
|  |  |  |  |  |  |  |  |  |  |
| **Risk Status** |  |  |  |  |  |  |  |  |  |
| Standard | 156 (58%) | 29 (52%) | 14 (74%) | 22 (47%) | 20 (63%) | 27 (56%) | 39 (68%) | 5 (63%) | .227 |
| High | 111 (42%) | 27 (48%) | 5 (26%) | 25 (53%) | 12 (38%) | 21 (44%) | 18 (32%) | 3 (38%) |  |
|  |  |  |  |  |  |  |  |  |  |
| **Hydrocephalus** |  |  |  |  |  |  |  |  |  |
| No | 114 (46%) | 13 (25%) | 7 (41%) | 19 (41%) | 19 (59%) | 25 (56%) | 25 (50%) | 6 (75%) | .**008** |
| Yes | 136 (54%) | 39 (75%) | 10 (59%) | 27 (59%) | 13 (41%) | 20 (44%) | 25 (50%) | 2 (25%) |  |
| Unknown | 17 | 4 | 2 | 1 | 0 | 3 | 7 | 0 |  |
|  |  |  |  |  |  |  |  |  |  |
| **VPS** |  |  |  |  |  |  |  |  |  |
| No | 191 (78%) | 39 (80%) | 15 (83%) | 30 (65%) | 27 (84%) | 30 (67%) | 43 (90%) | 7 (88%) | .**047** |
| Yes | 55 (22%) | 10 (20%) | 3 (17%) | 16 (35%) | 5 (16%) | 15 (33%) | 5 (10%) | 1 (13%) |  |
| Unknown | 21 | 7 | 1 | 1 | 0 | 3 | 9 | 0 |  |
|  |  |  |  |  |  |  |  |  |  |
| **Latency to RT (days)** |  |  |  |  |  |  |  |  |  |
| Mean (SD) | 44.3 (33.1) | 44.5 (27.8) | 42.7 (21.1) | 39.6 (28.0) | 42.8 (19.8) | 37.4 (11.3) | 54.2 (52.9) | 43.8 (27.7) | .403 |
| <42 | 160 (63%) | 33 (60%) | 10 (77%) | 34 (72%) | 20 (65%) | 29 (67%) | 31 (54%) | 3 (50%) |  |
| 42+ | 92 (37%) | 22 (40%) | 3 (23%) | 13 (28%) | 11 (35%) | 14 (33%) | 26 (46%) | 3 (50%) |  |
| Unknown | 15 | 1 | 6 | 0 | 1 | 5 | 0 | 2 |  |
|  |  |  |  |  |  |  |  |  |  |
| **Radiation** |  |  |  |  |  |  |  |  |  |
| Photon | 139 (53%) | 23 (41%) | 19 (100%) | 25 (53%) | 14 (48%) | 48 (100%) | 9 (16%) | 1 (25%) | ^<^.**001** |
| Proton | 121 (47%) | 33 (59%) | 0 (0%) | 22 (47%) | 15 (52%) | 0 (0%) | 48 (84%) | 3 (75%) |  |
| Unknown | 7 | 0 | 0 | 0 | 3 | 0 | 0 | 4 |  |
|  |  |  |  |  |  |  |  |  |  |
| **CSI Dose** |  |  |  |  |  |  |  |  |  |
| ≥30Gy | 182 (78%) | 34 (87%) | 8 (67%) | 40 (85%) | 20 (71%) | 44 (100%) | 29 (51%) | 7 (100%) | ^<^.**001** |
| <30Gy | 52 (22%) | 5 (13%) | 4 (33%) | 7 (15%) | 8 (29%) | 0 (0%) | 28 (49%) | 0 (0%) |  |
| Unknown | 33 | 17 | 7 | 0 | 4 | 4 | 0 | 1 |  |
|  |  |  |  |  |  |  |  |  |  |
| **Concurrent Chemo** |  |  |  |  |  |  |  |  |  |
| No | 171 (64%) | 40 (71%) | 13 (68%) | 34 (72%) | 19 (59%) | 43 (90%) | 19 (33%) | 3 (38%) | **^<^.001** |
| Yes | 96 (36%) | 16 (29%) | 6 (32%) | 13 (28%) | 13 (41%) | 5 (10%) | 38 (67%) | 5 (63%) |  |
|  |  |  |  |  |  |  |  |  |  |
| **Concurrent Type** |  |  |  |  |  |  |  |  |  |
| None | 171 (64%) | 40 (71%) | 13 (68%) | 34 (72%) | 19 (59%) | 43 (90%) | 19 (33%) | 3 (38%) | **^<^.001** |
| Vincristine | 78 (29%) | 16 (29%) | 6 (32%) | 13 (28%) | 8 (25%) | 1 (2%) | 29 (51%) | 5 (63%) |  |
| Etoposide | 5 (2%) | 0 (0%) | 0 (0%) | 0 (0%) | 0 (0%) | 4 (8%) | 1 (2%) | 0 (0%) |  |
| Carboplatin and vincristine | 13 (5%) | 0 (0%) | 0 (0%) | 0 (0%) | 5 (16%) | 0 (0%) | 8 (14%) | 0 (0%) |  |
|  |  |  |  |  |  |  |  |  |  |
| **Adjuvant Chemo** |  |  |  |  |  |  |  |  |  |
| 0 | 98 (39%) | 7 (13%) | 6 (40%) | 21 (47%) | 7 (22%) | 41 (85%) | 15 (29%) | 1 (13%) | **^<^.001** |
| 1-2 | 37 (15%) | 8 (15%) | 2 (13%) | 2 (4%) | 22 (69%) | 1 (2%) | 1 (2%) | 1 (13%) |  |
| ≥3 | 118 (47%) | 39 (72%) | 7 (47%) | 22 (49%) | 3 (9%) | 6 (13%) | 35 (69%) | 6 (75%) |  |
| Unknown | 14 | 2 | 4 | 2 | 0 | 0 | 6 | 0 |  |
|  |  |  |  |  |  |  |  |  |  |
| **Adjuvant (Packer)** |  |  |  |  |  |  |  |  |  |
| 0 | 97 (42%) | 7 (14%) | 6 (40%) | 21 (54%) | 6 (26%) | 41 (85%) | 15 (30%) | 1 (17%) | **^<^.001** |
| 1-2 | 28 (12%) | 8 (16%) | 2 (13%) | 0 (0%) | 16 (70%) | 1 (2%) | 1 (2%) | 0 (0%) |  |
| ≥3 | 107 (46%) | 36 (71%) | 7 (47%) | 18 (46%) | 1 (4%) | 6 (13%) | 34 (68%) | 5 (83%) |  |
| Unknown | 35 | 5 | 4 | 8 | 9 | 0 | 7 | 2 |  |

**Table S2: Characteristics of patients receiving adjuvant chemotherapy**

|  | No adj chemo | $\geq1 cycle adj chemo$ | $\geq3 cycles adj chemo$ |
| --- | --- | --- | --- |
| Age at Dx  *(median*) | 30.5 yrs | 26.5 yrs | 25.5 yrs |
| KPS (*median)* | 80.0 | 80.0 | 80.0 |
| Maximal tumor dimension (*cm, median*) | 4.2 cm | 4.1 cm | 4.3 cm |
| M-Stage>1 (%) | 11% | 20% | 22% |
| Days to RT (*median)* | 42.0 days | 35.0 days | 34.5 days |
| Concurrent chemo | 11% | 51% | 55% |

**Figure S1: Subgroup analyses – PFS**

Progression-free survival was improved with adjuvant chemotherapy in patients with M0 (A) but not M1-3 (B) disease. Progression-free survival was unchanged in patients with STR (C) or GTR (D) who received adjuvant chemotherapy. Progression-free survival was unchanged in patients treated with low dose craniospinal radiation who received or did not receive adjuvant chemotherapy (E). P-values are calculated by Log-Rank test.

**Figure S2: Subgroup analyses – molecular subtypes**

Because all WNT-activated tumors were treated with adjuvant chemotherapy, no analysis could be performed for this subgroup. SHH-activated tumors showed no change in survival (A) but improved PFS with adjuvant chemotherapy (B). Non-SHH/non-WNT tumors showed improved survival (A) but no change in progression-free survival with adjuvant chemotherapy (B). All WNT-activated tumors got standard dose craniospinal radiation and no subanalysis could be performed. SHH-activated tumors showed no change in survival (E) but improved progression-free survival (F) with standard dose craniospinal radiation. Non-SHH/Non-WNT activated tumors showed no differences in either survival (G) or progression-free survival (H) depending on craniospinal radiation dose. P-values are calculated by Log-Rank test.
